# Nociplastic pain: the prominent role of non-neuronal cells in central and peripheral sensitization

**DOI:** 10.3389/fimmu.2026.1677310

**Published:** 2026-01-27

**Authors:** Weidong Lai, Xin Tang, Sijia Chen, Yihan Zhou, Zihan Qi, Menglei Cao, Cunrui Yuan, Jie Yu, Chengping Wen

**Affiliations:** School of Basic Medical Science, Zhejiang Chinese Medical University, Hangzhou, China

**Keywords:** central sensitization, chronic pain, nociplastic pain, non-neuronal cells, synaptic plasticity

## Abstract

Nociplastic pain (NPP) is a recently defined form of chronic pain, characterized by altered nociception in the absence of clear evidence of nociceptive or neuropathic pain. It is believed to be driven by maladaptive changes in nociceptive processing, mediated by the peripheral and/or central nervous system sensitization. However, the exact pathogenic basis for NPP initiation and development remains unclear, and the potential clinical manifestations and biomarkers lack a clear classification. Emerging evidence now emphasizes the role of complex neuron–non-neuronal cell interactions, highlighting non-neuronal cells (e.g., circulating immune cells and glial cells) in NPP pathogenesis. In this review, we delineate the key conceptual distinctions of NPP within the spectrum of chronic pain and present the latest evidence outlining how non-neuronal cells mediate NPP progression via peripheral and central sensitization, as well as the associated synaptic plasticity mechanisms. We also summarize current and experimental therapeutic approaches explored in preclinical and clinical studies of NPP. We hope that this review provides a theoretical foundation for subsequent analgesic target screening and the drug development for NPP.

## 1 Introduction

According to the International Association for the Study of Pain (IASP), chronic pain (CP) is an unpleasant sensory and emotional experience associated with actual or potential tissue damage. It imposes a substantial economic burden and poses significant threats to both the mental and physical well-being of individuals (1). In clinical practice, researchers have previously identified a new type of pain mechanism that is independent of persistent inflammation (nociceptive pain, NCP) and/or) tissue damage and nerve injury (neuropathic pain, NP). Thus, in order to further clarify the boundaries between these pain types, the international community of pain researchers has proposed a novel mechanism—NPP (2). This independent mechanism is described as “pain that arises from altered nociception despite no clear evidence of actual or threatened tissue damage causing the activation of peripheral nociceptors or evidence for disease or lesion of the somatosensory system causing the pain”. This concept appears to successfully include the patients who exhibit neither obvious activation of nociceptors, nor neuropathy (defined as disease or damage of the somatosensory system), yet whose clinical and psychophysical findings suggest altered nociceptive function (3).

When applying this classification in clinical practice, it remains considerably challenging. Clinically, it is not uncommon to encounter patients with NPP who also have overlapping features of NP and/or) NCP. A large number of patients, who do not fully meet the criteria for NP and NCP have often been broadly categorized as having NPP by the researchers. In 2021, to further clarify NPP, the IASP defined clearer diagnostic boundaries and criteria for NPP-related chronic pain, including: “I. pain duration ≥3 months; II. a regional rather than discrete distribution; III. the pain that cannot be entirely be explained by nociceptive or neuropathic mechanisms; and IV. clinical signs of pain hypersensitivity are present in the region of pain. A history of pain hypersensitivity in the affected region of pain and defined comorbidities strengthen the probability of nociplastic pain, and both must be present to designate probable nociplastic pain” (4).

With an estimated prevalence ranging from 5% to 15% of the general population, NPP mainly manifests as distinct neurological symptoms in local or multiple regions of the body (5), such as burning sensation, soreness, and heaviness, which may be spontaneous or induced by factors such as climate and emotions (6). Previous studies have commonly applied the biopsychosocial model to explain the pathogenic factors of NPP (7), including psychological, emotional, sexual, and physical abuse, or their combination. Family history of pain experience also appears to be associated with NPP (8). However, the clear etiology and pathogenesis of NPP still have not yet been systematically clarified yet.

**Figure 1:**
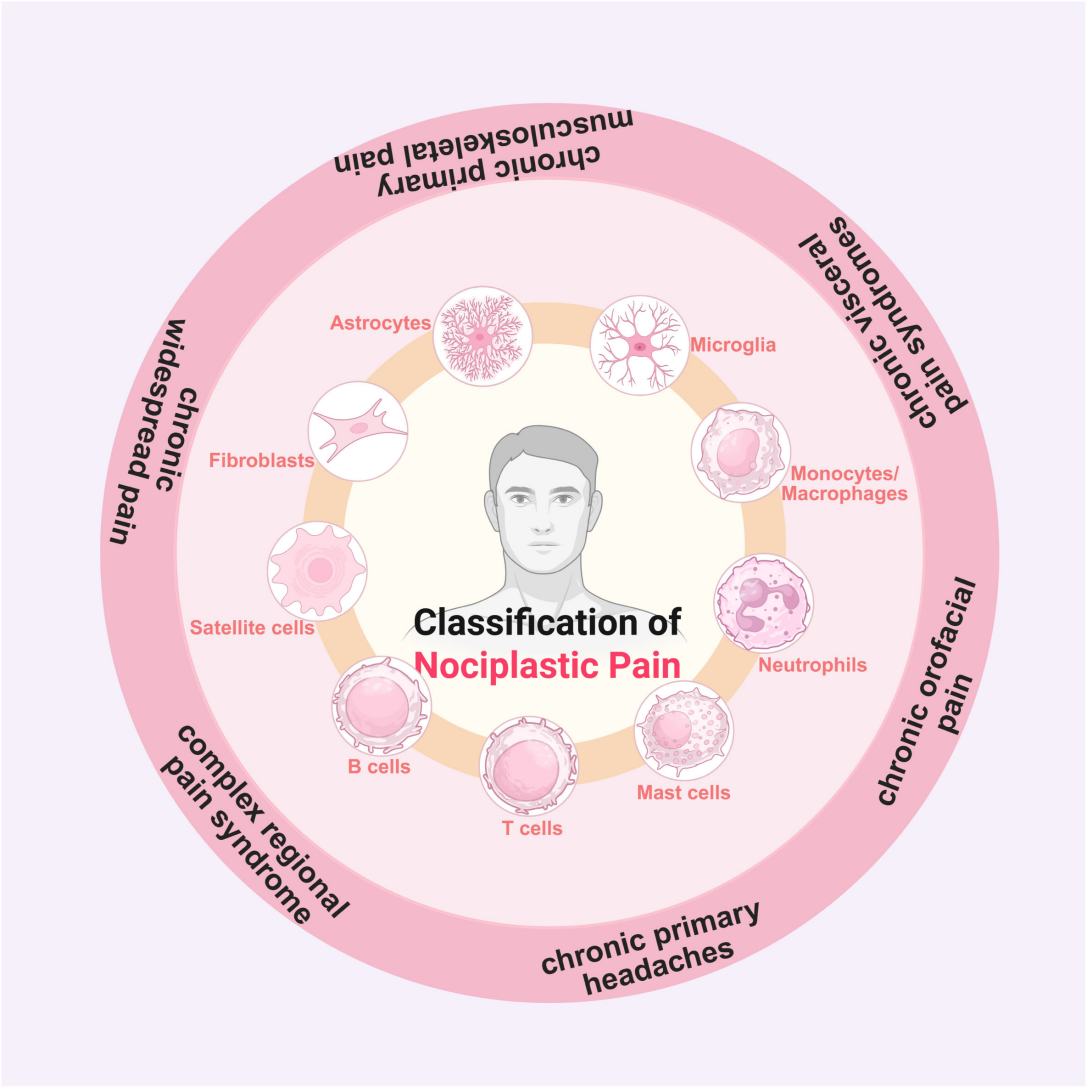
Classification and pathogenesis of nociplastic pain. Chronic widespread pain, CRPS, chronic primary headaches and orofacial pain, chronic visceral pain syndromes, and chronic primary musculoskeletal pain are six kinds of typical forms of NPP in clinical practice. Non-neuronal cells such as astrocytes, microglia, satellite glial cells, T cells, B cells, mast cells, neutrophils, and monocytes/macrophages contribute to NPP development.

Sensitization, particularly central sensitization, may serve as a common mechanistic link between the pathogenesis of NPP and that of NP and/or) NCP. This may explain the coexistence of multiple pain mechanisms within an individual’s pain state. NPP often overlaps with the neuropathic pain due to shared underlying pathological mechanisms, especially central sensitization (9). Changes in central synaptic plasticity, which are involved in regulating the development and maintenance of chronic pain, are considered a primary cause of central sensitization in NPP (10). Accordingly, several conditions related to central sensitization, such as chronic widespread pain, fibromyalgia (FMS), irritable bowel syndrome (IBS), chronic primary low back pain (CPLBP), complex regional pain syndrome (CRPS), chronic primary headaches and orofacial pain, chronic visceral pain syndromes, and chronic primary musculoskeletal pain, etc., are generally classified under NPP (**Figure 1**). The mechanisms of central sensitization are rely on the pathological changes, particularly alterations in synaptic plasticity changes, within the central nervous system. However, it remains unclear how synaptic plasticity mediates central sensitization and subsequently induces NPP, as well as which pathological factors drive changes in synaptic plasticity.

Non-neuronal cells, including circulating and central immune cells, have been implicated in the modulation of chronic pain through the regulation of central sensitization (11). However, the role of non-neuronal cells in the development and maintenance of NPP remains uncertain. Previous studies have shown that non-neuronal cells can regulate central sensitization by modulating synaptic plasticity (12, 13). Similarly, whether non-neuronal cells can also influence NPP through the regulation of synaptic plasticity and central sensitization requires further clarification.

In this review, we aim to examine the relationship between NPP and non-neuronal cells, with a particular emphasis on central glial cells and circulating immune cells, and to elucidate the underlying mechanisms through which non-neuronal cells regulate NPP.

## 2 Materials and methods

In this review, two independent investigators were involved in the literature search (**Table 1**) and screening process based on predefined inclusion and exclusion criteria. The search covered four databases—PubMed, EMBASE, Web of Science, and the Cochrane Library—from their inception to the present. To ensure retrieval of comprehensive literature retrieval, we employed both central concepts and their established subordinate terms. The keyword “nociplastic pain” was represented by specific conditions, including fibromyalgia (FMS), irritable bowel syndrome (IBS), complex regional pain syndrome (CRPS), and chronic primary low back pain (CPLBP). Similarly, the concept of “non-neuronal cells” was expanded to include immune cells and glial cells. Furthermore, according to Medical Subject Headings (MeSH), the terms “nociplastic pain,” “central sensitization,” and “synaptic plasticity” were employed in the literature search. These terms were combined using Boolean operators and adapted to the specific query syntax of each database. Inclusion criteria: 1. Publications limited to the English language. 2. All types of studies were considered, including clinical research, preclinical studies, reviews, systematic reviews, and meta-analyses. Exclusion criteria: Literature unrelated to nociplastic pain. Literature unrelated to non-neuronal cells. Literature unrelated to neural or synaptic plasticity. Publications or books that could not be accessed due to being outdated or unavailable.

**Table 1:** Searching strategy.

Search number	Search terms
Search #1	"Nociplastic pain" OR "NPP" OR "Fibromyalgia" OR "Irritable Bowel Syndrome" OR "Chronic widespread pain" OR "Chronic primary headaches" OR "Chronic primary orofacial pain" OR " Chronic visceral pain syndromes"
Search #2	"Non-neuronal cells" OR "Monocytes" OR "Macrophages" OR "T cells" OR "B cells" OR "Neutrophils" OR "Mast cells" OR "Satellite cells" OR "Central glial cells" OR "Microglia" OR "Astrocytes" OR "Oligodendrocytes"
Search #3	"Synaptic change" OR "Synapse modulation" OR "Synaptic plasticity" OR "Plastic change"
Search #4	Search #1 and Search #2 and Search #3

## 3 Potential pathogenic basis related to NPP initiation and development

### 3.1 Pre-existing virus infections

Viral infections usually induce NP or NCP through direct damage to neural tissues or inflammation. However, a subset of patients who have undergone antiviral or anti-inflammatory therapy, and in whom clear evidence of neural tissue damage is not apparent, continues to experience pain. These cases are now defined as NPP. Accordingly, the early inflammatory stage of rheumatoid arthritis can be classified as NCP. However, after receiving 3 months of treatment, when the inflammation has declined while the recurrent pain persists, the condition may be better explained by NPP (14). Similarly, chronic inflammatory pain caused by pathogen infection can be regarded as NCP, whereas chronic pain that persists even after the inflammation subsides should be classified as NPP. Although numerous studies have shown that some viral infections can lead to peripheral neuropathy, there are some patients who continue to experience pain even after receiving standard first-line anti-inflammatory and analgesic therapies in clinical practice (15, 16). For example, in the synovial fluid of 38 patients with persistent joint pain following chikungunya virus infection, neither the virus itself nor the expression of its corresponding proteins, mRNA, or pro-inflammatory cytokines was detectable, suggesting that joint pain can occur independently of active viral infection or local inflammation (17). In 2021, Maria M ZanoneMaria M. Zanone et al. reported that, among the 94 hepatitis C virus (HCV)-infected patients who had received direct-acting antiviral treatment, 37 patients developed chronic pain. Although the authors referred to this pain as neuropathic pain, this classification may not be appropriate. In the absence of reported nerve damage or lesions, these cases might be more accurately defined as NPP-related chronic pain (18).

Pre-existing infections may represent one of the potential pathogenic factors involved in NPP initiation and development. For example, A. Baroni et al. showed that nearly one-third of post-COVID-19 patients experienced late-onset pain, and this reduction in pain threshold reduction was interpreted as central sensitization (19). In 2024, Man Ting Au et al. retrospectively reviewed two patients who had been affected by COVID-19; both suffered from knee joint pain after recovery. Moreover, a decreased paw withdrawal threshold decrease was observed in mice injected with viral spike receptor-binding domain (RBD), and this hypersensitivity, as well as the chronic pain–related secretion of substance P (SP), could be successfully reversed by the endothelin receptor blocker macitentan (20). Studies have also reported that FMS can be induced or triggered following different types of viral pre-infection (21, 22). These findings suggest that the pre-existing infection may act as an inducing factor, with pathological changes in the nervous system being more likely responsible for sustained pain.

Although NPP may not be a direct indicator of the outcome of viral pre-infection, we still believe that a potential connection appears to exist. We speculate that the unrevealed pathological changes in the nervous system following pre-infection may represent one of the potential mechanisms underlying mechanisms contributing to NPP. For example, microorganisms have been shown to directly interact with components of the blood–brain barrier (BBB), leading to coagulopathy and tight junction disruption, inducing lung inflammation that results in hypoxia, and ultimately increasing BBB permeability (23). It has also been reported that SARS-CoV-2 RNA can be detected in cerebrospinal fluid alongside abnormal brain magnetic resonance imaging findings in COVID-19 patients (24). These pathological mechanisms provide a basis for viral invasion of the central nervous system, which may represent a latent risk factor for NPP initiation of NPP.

**Figure 2:**
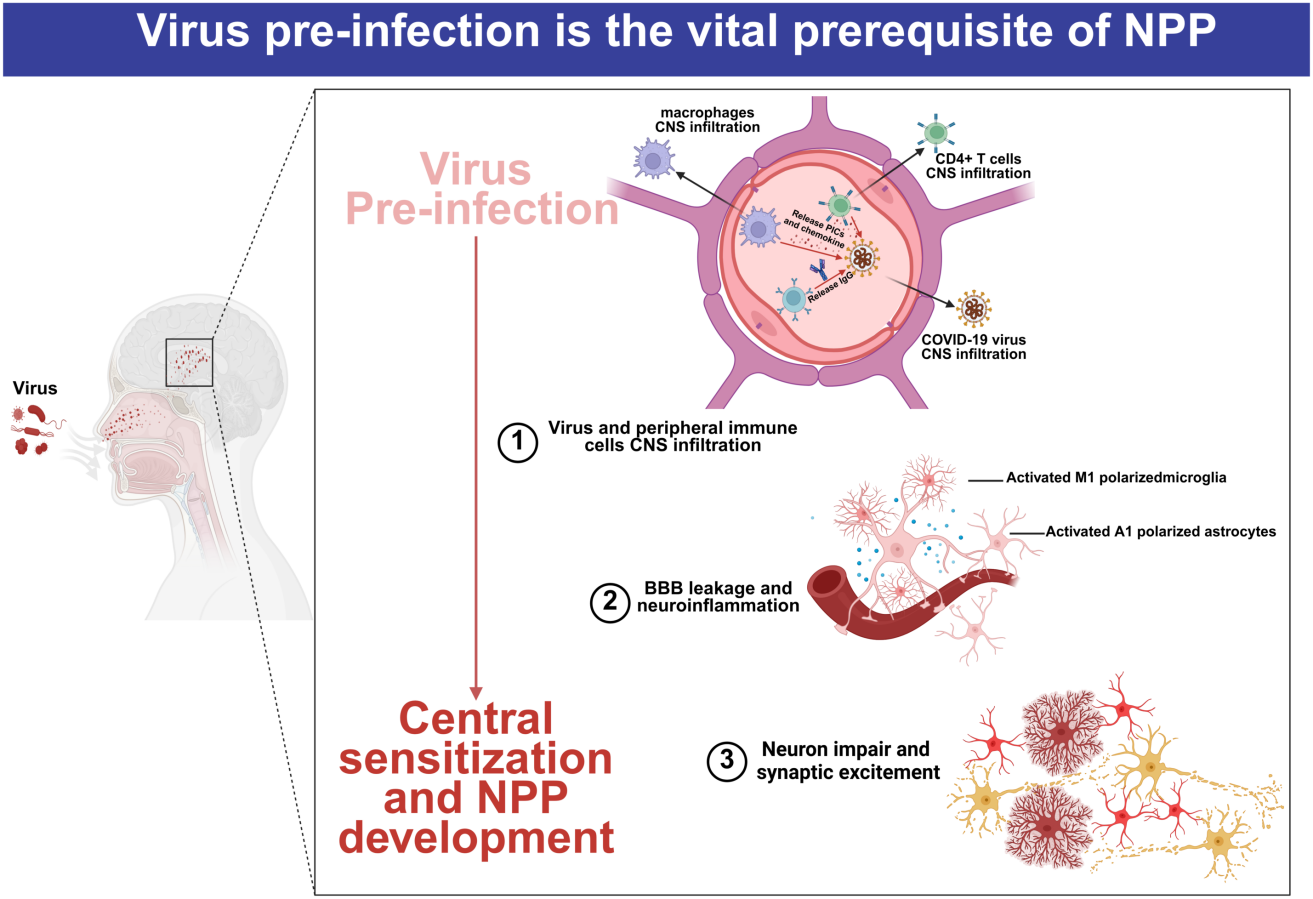
Pathogens pre-infection as a vital prerequisite for NPP development. In the early stage, the pathogenic microorganisms can successfully invade into the human body through the respiratory tract or digestive tract, respectively. In addition to triggering immune responses mediated by immune cells, these pathogens can disrupt the blood–brain barrier and enter into the CNS, thereby activating and polarizing central glial cells. Pathogen invasion increases blood–brain barrier permeability and induces central inflammation, ultimately leading to nerve damage and changes in synaptic excitability. Upon repeated stimulation, long-term chronic inflammatory signaling induces central sensitization, promoting the occurrence of NPP.

In addition, the central invasion and infiltration of immune cells following viral pre-infection may serve as a trigger for NPP. Studies have shown that human immunodeficiency virus 1 (HIV-1)-infected leukocytes not only secrete matrix metalloproteinases (MMP)-2 and MMP-9 that impair the BBB, but also promote immune cell infiltration into the central nervous system via chemokine C-C motif ligand 2 (CCL2) (25). Bente Winkler et al. found that macrophages, derived from the peripheral blood, invaded across the BBB mediated via BBB-forming glial cells and promoted neuronal injury following brain inflammation (26). They further reported that central inflammation attracted more immune cells to cross the BBB than viral brain invasion alone. After infection with the porcine hemagglutinating encephalomyelitis virus, Jing Zhang et al. observed that circulating monocytes/macrophages crossed the BBB into the central nervous system in model mice, recruited by activated microglia (27). In addition, in the collagen-induced arthritis (CIA) mouse models, CD4^+^ T cells were shown to cross the BBB and induce central nervous system inflammation through activating microglial activation, indicating that immune cell invasion may participate in NPP development following pre-infection (**Figure 2**). However, whether the changes in circulating immune cells or even other non-neuronal cells exert subsequent long-term effects on the nervous system, after viral pre-infection remains unclear (28).

### 3.2 Genetic predisposition

Studies indicate that the familial association rate for FMS is approximately between 12%–21% (roughly 1 in 5 to 1 in 9) (29). Concurrently, several genetic loci have been implicated in FMS susceptibility, including the *catechol-O-methyltransferase (COMT)* gene, the serotonin transporter-linked polymorphic region (5-HTTLPR) of the *SLC6A4* gene, as well as *DRD2* and *DRD4*, which encode dopamine receptors (30). In a separate genome-wide analysis, Keira J. A. Johnston et al. investigated six chronic overlapping pain conditions (COPCs)—CPLBP, endometriosis, temporomandibular joint disorder, IBS, and broad headache—and identified 24 independent single-nucleotide polymorphisms (SNPs) significantly associated with nociceptive pain, implicating 127 unique genes. These findings strongly suggest a genetic contribution to NPP, with a liability-scale SNP-based heritability (h²) of 0.025 (SE = 0.0014) (31). In addition, the variation in the transient receptor potential vanilloid 1 (TRPV1) gene has also been shown to be associated with NPP (32).

### 3.3 Alcohol abuse and gut microbiota dysbiosis

Long-term alcohol use disorder induces plastic adaptations that subsequently contribute to the development of negative affective states, such as anxiety and depression, as well as the manifestation of hyperalgesia (33, 34). An imbalance in gut microbiota, characterized by a reduced ratio of beneficial to harmful bacteria, represents a significant exposure factor in the development of NPP. A recent meta-analysis encompassing 23 studies with 1,340 subjects revealed that patients with IBS exhibited significantly lower fecal levels of Lactobacillus and Bifidobacterium, alongside significantly higher levels of Escherichia coli and marginally elevated Enterobacter. Supporting a causal role, fecal microbiota transplantation from FMS model mice into healthy recipients induced hyperalgesia in the recipient animals (35). Conversely, transplantation of gut microbiota from healthy female donors to female FMS patients alleviated their hyperalgesia (36).

### 3.4 Specific alternation of rhythm in temperature

Rapid and frequent alternation of specific temperature rhythms is an important factor contributing to reduced sensitivity of the sympathetic nerves (37). In rodent models, a temperature of 4°C is sufficient to induce long-term abnormal pain, whereas at 6°C, corresponding long-term pain cannot be successfully induced (38). Under repeated subthreshold cold stimulation administered at 30-min intervals, organisms lacking local glutamatergic/sympathetic neural warning systems exhibit disrupted glutamate (Glu) secretion, alongside sterile frostbite-like injury of peripheral nociceptors (39). At present, several established animal models exist for FMS. Among them, temperature rhythm alternation models mainly include the intermittent cold stress (ICS) model and the thermal grill illusion (TGI) model (40). From a neurobiological perspective of neurobiology, the ICS animal model can largely replicates the clinical characteristics and complications of FMS, suggesting that repeated and intermittent exposure to excessively high or low temperatures may represent a pathogenic factor in NPP.

### 3.5 Gender duality

Gender duality is also observed in NPP-related chronic pain. According to a review published in The Lancet in 2021, NPP is present in four conditions: CPLBP, FMS, chronic temporomandibular disorders (TMD), and IBS. The female-to-male incidence ratio is approximately 2:1, while the incidence of CPLBP of unknown causes varies depending on the study region. For chronic primary bladder pain syndrome, the ratio is approximately 2–10:1, and for chronic primary pelvic pain syndrome, the ratio in men and women is reported as 0:1 and 1:0, respectively (3). In rodent studies, the decrease in mechanical pain threshold after ovary removal was similar to that observed in intact female mice, suggesting an important role of estrogen in the ICS-induced NPP model (39). Interestingly, research conducted by Włodzimierz Samborski et al. demonstrated that, compared with healthy controls, there were no significant differences in estradiol and progesterone levels in patients with FMS. This suggests that either estrone, estradiol, or other types of estrogen subtypes may mediate the onset of NPP (41).

## 4 Clinical manifestations and potential biomarkers

### 4.1 Blood glucose

José V. Pardo et al. reported that, under intermittent cold stimulation (ICS) conditions, compared with 11 healthy female controls, the blood glucose levels in 13 female patients with fibromyalgia (FMS) were decreased, suggesting that changes in blood glucose may serve as a potential predictive indicator for the clinical detection of NPP. However, there is a minor drawback: the researchers observed fluctuations in blood glucose levels during intermittent cold and heat stimulation but did not find a significant increase in pain sensitivity in FMS patients after either stimulation. This may be due to the use of pain-relieving medications by the patients, which could reduce their sensitivity to pain induction (42). Approximately 30% of female FMS patients exhibit a reduced adrenocorticotropic hormone (ACTH) and epinephrine response to hypoglycemia. This phenomenon is associated with dysfunction of the hypothalamic–pituitary–adrenal (HPA) axis in FMS patients. When the degree and duration of hypoglycemia were precisely controlled using the low-glycemic–high-insulin clamp technique, the ACTH response in FMS patients after a single insulin injection was reduced by 30% compared with healthy subjects (43). Moreover, low levels of epinephrine and adrenocorticotropic hormone further impair the ability to increase blood glucose through a positive feedback loop (44). This phenomenon may be related to sympathetic inactivation caused by frequent internal or external stimulation, which reduces adrenaline secretion in the body, thereby inhibiting the secretion of neuropeptide Y (a marker of chronic sympathoadrenal activity) and suppressing the adrenaline-mediated increases in blood glucose (45). This clinical manifestation of hypoglycemia may serve as an important indicator for assessing whether the HPA axis imbalance in patients with NPP.

### 4.2 Insulin-like growth factor levels

A large-scale meta-analysis including 512 patients with FMS and 308 healthy subjects, showed that the level of IGF-1 levels in patients with FMS were significantly reduced (46). Pulsatile growth hormone (GH), secreted by the anterior pituitary gland, stimulates the hepatic synthesis and secretion of IGF-1; therefore, reduced IGF-1 secretion may be attributed to the imbalance of the hypothalamus–growth hormone–IGF-1 axis (43). Moreover, studies have shown that aging and obesity are positively correlated with low IGF-1 expression (47). Thus, reduced IGF-1 secretion may be associated with NPP.

### 4.3 ACTH and substance P

After exogenous insulin injection, an exaggerated ACTH response of the ACTH in the patients was successfully induced in patients (48). In CPLBP, its ACTH levels were also significantly increased compared with those in control groups (49). HPA axis impairment and hyperresponsiveness of ACTH release may therefore represent significant features of NPP. In a clinical trial involving 30 female patients with FMS, increased secretion level of SP in the cerebrospinal fluid (CSF) was observed in the cerebrospinal fluid (CSF) of all the FMS patients (50). I J RussellRussell et al. further reported that, compared with 30 healthy controls, a threefold higher concentration of SP was detected in the CSF of all 32 FMS patients (51). Moreover, in the CSF of FMS patients, increased SP expression was positively correlated with smoking, while the SP expression was independent of both calcitonin gene-related peptide (CGRP) and beta-endorphin levels, suggesting that SP may represent another potential biomarker (52, 53).

## 5 Circulating immune cells may induce NPP development

### 5.1 Monocytes/macrophages

Macrophages, derived from monocytes migrating out of blood vessels, play a crucial role in innate immunity (54). Recent studies have reported macrophage infiltration in the trigeminal nerve tissues in FMS model mice, suggesting that monocytes/macrophages are closely related to NP (55). However, how monocytes/macrophages contribute to NP remains unclear. It has not been clarified yet. It was shown that treatment with dried powdered ginger rhizome improved both allodynia and hyperalgesia by decreasing the inflammatory responses mediated by nitric oxide (NO), prostaglandin E2 (PGE2), thromboxane B2 (TXB2), and interleukin-1β (IL-1β) in lipopolysaccharide (LPS)-stimulated macrophages in FMS model mice, indicating that macrophages may contribute to NPP through the release of pro-inflammatory mediators (56). However, A. Kashipaz et al. argued that cytokine dysregulation of cytokines by CD14-positive circulating monocytes may not be a predominant factor in FMS pathogenesis of FMS (57). Mario D. Cordero et al. reported that coenzyme Q10 levels and mitochondrial membrane potential in monocytes were decreased, alongside increased mitochondrial superoxide levels, suggesting that NPP may be closely related to mitochondrial autophagy in monocytes (58).

During the progression of CRPS to full-blown FMS, CD68-positive monocytes/macrophages play a key role in local infiltration of muscle, dorsal root ganglia (DRG), and other spinal cord sites, increasing macrophage numbers to drive neuronal plasticity and enhance NPP (59). Juan J García et al. found that the macrophages released higher serum concentrations of inflammatory chemokines, such as macrophage-derived chemokine (MDC)/CCL22, promoting NPP by regulating immune cell migration and local infiltration (60).

### 5.2 Neutrophils

Neutrophils also play an irreplaceable role in NPP development. Regarded as a common inflammatory cells, migration and recruitment of neutrophils to the injured sites result in the release of pro-inflammatory mediators, and the phagocytosis of both pathogens and tissue debris (61). Undoubtedly, the pro-inflammatory and pain-inducing effects of neutrophils are well established; however, there are still few direct reports explaining how neutrophils induce NPP through these pro-inflammatory functions. Sara Caxaria et al. showed that adoptive transfer of neutrophils from FMS patients conferred mechanical pain to recipient naïve mice, indicating a pathogenic role of neutrophils in NPP. Neutrophils were also shown to infiltrate the dorsal root ganglia (DRG), suggesting that neutrophil infiltration into the DRG may be a critical contributor to NPP (62). In addition, adhesion and recruitment of neutrophils were enhanced in FMS patients, promoting infiltration into inflammatory sites in FMS patients.

Regarding neutrophil proportions, it has been reported that the neutrophil-to-lymphocyte ratio (NLR) is increased in patients with FMS. That is, within the inflammatory microenvironment of FMS, neutrophil numbers are elevated (63). Patients with FMS and high NLR values also exhibited higher scores on the revised FMS questionnaire impact scale. Furthermore, in IBS patients, the NLR levels were similarly elevated in patients with IBS compared with control groups. Together, these findings indicate that high NLR may be positively associated with NPP (64, 65). However, given the variability of NLR levels among FMS patients with FMS, some studies do not support the use of NLR as a reliable neutrophil-related indicator (66, 67). Subsequent investigations demonstrated that elevated NLR in FMS patients correlates with variations in hypersensitive C-reactive protein levels, suggesting that NLR instability may reflect fluctuations in disease activity (68). Hilal Telli et al. further reported that, in female gender, patients with FMS, high NLR levels were accompanied by higher Visual Analog Scale (VAS) rest scores, indicating a positive association between elevated NLR and NPP (64).

### 5.3 Mast cells

NPP is widely reported to be correlated with mast cell activation. Local infiltration and migration of mast cells, leading to sensitization of nearby nociceptive afferents, are considered vital contributors to NPP pathogenesis (69).

In FMS patients with FMS, skin biopsy analyses have revealed increased numbers of mast cells (70). In the intestinal tissues of patients with IBS, mast cells infiltrate into the posterior ganglionic fibers of the sympathetic nerves, peptidergic nerve fibers, vagal nerve fibers, vagus nerve fibers and the periphery of intestinal neurons, providing an anatomical basis for neuroimmune interactions (71). Notably, approximately 70%–84% of patients with IBS exhibit local mast cell infiltration of mast cells in their intestinal tissues. Moreover, the phenomenon of mast cell infiltration surrounding intestinal nerves occurs approximately three times more frequently in IBS patients than in controls, and the number of degranulated mast cells is increased by approximately 1.5-fold (72). Through synapse-like structures, mast cells can directly contact non-cholinergic and non-adrenergic nerves and mediate neuroimmune signaling via adhesion molecules such as monocyte chemotactic protein-1 (MCP1) and N-cadherin (71, 73). In addition, chemokine secretion facilitates local infiltration of mast cell infiltration. Zhao et al. reported increased serum levels of MCP1/CCL2 in patients with FMS patients (74). CCL2 can bind to its receptor CCR2 on peripheral nerve terminals, thereby inducing NPP (75).

Beyond infiltration and migration, mast cell degranulation also contributes to NPP development. Evelyne da Silva Brum et al. observed mast cell infiltration in FMS model mice, accompanied by mechanical and cold allodynia as well as muscle fatigue. Following administration of compound 48/80 or the mast cell membrane stabilizer ketotifen to deplete mast cell activity, mechanical and cold allodynia were successfully reversed, supporting a pathogenic role of mast cell degranulation in NPP (76). In water avoidance stress model rats, treatment with cromolyn sodium or the neutralizing agent of nerve growth factor–neutralizing agents significantly inhibited synaptic plasticity in the mesenteric region and alleviated stress-induced abdominal pain induced by stress, suggesting that mast cell degranulation effect of mast cells, together with nerve growth factor (NGF), induces synaptic plasticity changes leading to abdominal pain (77). Mast cells release histamine and proteases during degranulation. Kayo Masuko et al. demonstrated that through the degranulation effect of mast cells, the levels of mast cell–derived serine protease tryptase levels were also increased following mast cell degranulation, which subsequently activated proteinase-activated receptor-2 (PAR-2) signaling pathways to modulate hypersensitivity (78). Histamine H1 and histamine H2 can respectively act on their respective receptors on the visceral afferent nerves and intestinal neurons, whereas histamine H3 receptors mediate the inhibition of rapid synaptic transmission in intestinal neurons. PAR-2 expressed on the intestinal nerves and afferent nerves induces neuronal excitation of intestinal neurons (79–81). Furthermore, the activation of the 5-hydroxytryptamine receptor 1A (5-HT1A), rather than the histamine receptors, has also been implicated in the complex abdominal pain mediated by mast cell degranulation (82).

Ramona D’Amico et al. demonstrated that the P2X7 receptor (P2X7R)/NLRP3 signaling pathway is involved in mast cell degranulation. Use of the P2X7R antagonist Brilliant Blue G (BBG) prevented NLRP3 inflammasome activation and subsequent release of interleukin (IL)-1 and IL-18, thereby reducing FMS symptoms (83). Pio Conti reported that mast cell degranulation effects, mast cell releases inflammatory mediators that induce low-grade inflammation and trigger the onset of FMS (70). Parallelly, similar mechanism was found in IBS (84). Ivashkin et al. reported that in the serum of IBS patients, the expression of pro-inflammatory cytokines like IL-1, IL-2, and TNF-α were increased significantly (85), which could achieve to NPP through impairing intestinal barrier, inhibiting the secretion of neurotransmitters and maintaining chronic low-grade inflammation (86). Therefore, receptors such as PAR-2, 5-HT2A, 5-HT3, H1, NK1, P2X7R, and MrgprB2 may represent promising therapeutic targets for regulating abnormal mast cell activity in NPP.

### 5.4 T lymphoid cells

As a typical representative population of adaptive immune cells, T cells play an important role in NPP induction, and numerous studies have reported associations between T cells and NPP. T cells, as well as interleukins released by lymphocytes, can induce NPP formation of NPP (87). Hiroshi Ueda et al. observed allodynia in recipient mice following adoptive transfer of purified CD4^+^ T cells, derived from splenocytes of acid-treated male mice, into naïve mice, indicating that CD4^+^ T cells participate in FMS pathogenesis (88). Chie Sugimoto et al. reported that the proportion of CD8^+^ mucosal-associated invariant T (MAIT) cells was increased in FMS patients with FMS and suggested this population as a potential biomarker (89). W. Hernanz et al. found that the numbers of CD69-positive (activation-induced marker) and CD25-positive (interleukin-2 receptor) positive T cells were decreased in FMS patients with FMS (90). However, Giuliana Guggino et al. did not identify significant differences in the proportion of T-cell subtype proportions between patients compared with FMS and healthy individuals. Interestingly, they observed upregulation of several cytokines secreted by CD4^+^ T cells, including IL-17A, IL-22, IL-9, TNF-α, and IFN-γ (91). Jessica L. Ross et al. demonstrated that pro-inflammatory cytokines, such as IL-1β released from T lymphocytes, could induce peripheral sensitization in an acid-sensing ion channel (ASIC)-dependent manner in somatosensory neurons (92, 93). In the cerebrospinal fluid (CSF) of FMS patients with FMS, Eva Kosek et al. reported elevated levels of IL-8, IL-1Ra, IL-4, and IL-10 were elevated (94). In contrast, D. J. Wallace et al. did not observe increased expression of cytokines such as IL-1β, sIL-2R, IFN-γ, TNF-α, IL-10, or IL-2 in FMS patients with FMS, but only detected increased IL-1Ra and IL-8 levels (95). In FMS model mice, the proportion of regulatory T helper cells (CD4^+^FOXP3^+^) was reduced, accompanied by increased IL-5 secretion from Th2 cells. Inhibition of IL-5 could successfully reversed mechanical hypersensitivity, suggesting that FMS is closely associated with T-cell dysregulation (96). In patients with FMS, imbalance of the Th1/Th2 cell ratio and excessive secretion of IL-8 and heat shock proteins are the prominent features. Studies have shown that physical exercise training can reduce the proportion of Th1 cells, rebalance the Th1/Th2 ratio, and inhibit the expression of IL-8 and heat shock protein expression, thereby alleviating pain and cognitive impairment in patients with FMS (97). N. Hader et al. identified a defect in CD4+ T cells, with reduced IL-2 secretion from CD4^+^ T lymphocytes was decreased (98). In patients with CRPS, Ines Kaufmann et al. reported that the ratio of CD8+ lymphocytes and the percentage of IL-2–producing T-cell subpopulations were lower than those in healthy donors, indicating a Th1/Th2 imbalance in NPP (99).

### 5.5 B cells

As an essential component of the immune system, B lymphocytes contribute to adaptive immunity by migrating to the injured sites and differentiating into plasma cells that release autoantibodies. In addition, B cells, as potent antigen-presenting cells, play an immunomodulatory role by releasing cytokines, activating auto-reactive T cells (100). Numerous studies have reported increased proportions of B cells elevated in NPP. Melissa E. Lenert et al. found increased numbers of MHC class II–positive B cells and activated plasma cells increased in peripheral blood, as well as elevated proportions of memory B cells was elevated in the bone marrow of female chronic widespread pain (CWP) model mice (101). Valentina Malafoglia et al. demonstrated that the proportion of μ-opioid receptor–positive B cells was lower than that in controls in blood samples from patients with FMS, suggesting that B cells μ-opioid receptor deficiency in B cells may impair endogenous opioid analgesic activity and contribute to sensitization, thereby supporting a pathological mechanism support for the aggravation of FMS and may prove the role for B-cell subsets in the pathogenesis of NPP (102).

Serena Fineschi et al. conducted transcriptomic analysis of B cells from 30 FMS patients with FMS and 24 healthy individuals and identified 60 potential differentially expressed genes. Among these, four genes with the most significant differences and interferon (IFN) responses—S100A8, VCAM, CD163, and ANXA1—were highlighted, indicating that B cells and their IFN secretion—IFN, are associated with FMS (103). Paulino Barragán-Iglesias et al. further showed that B cells can release type I IFN, which stimulates MNK-mediated eIF4E phosphorylation in dorsal root ganglion (DRG) neurons, leading to generate nociceptive sensitization and generalized pain (104). In addition, B cells secrete immunoglobulin G (IgG) as part of adaptive immunity. Through the B cell/IgG/Fc axis, IgG complexes can directly bind to the peripheral somatosensory neurons directly to induce neuropathic pain; however, whether this axis also induces NPP remains unclear (105). Eneström S. et al. identified dermal IgG deposits in the skin of FMS patients with FMS (106). Andreas Goebel et al. transferred IgG, donated from FMS patients with FMS into naïve mice and demonstrated that IgG interacted with satellite glial cells in the DRG to induce hypersensitivity to noxious mechanical and cold stimulation (107). Similarly, transfer of purified IgG isolated from CRPS patients with CRPS into control mice prolonged postsurgical hypersensitivity to noxious mechanical, cold, and heat stimulation (108).

## 6 Peripheral glial cells (satellite glial cells) may contribute to NPP development

Satellite glial cells (SGCs), the main cell type mediating myelin generation in the peripheral nervous system, surround sensory ganglion neurons in a circular arrangement at a distance of approximately 20 nm. Neurons and their synapses are wrapped within a single sheath via gap junctions, forming a discrete anatomical and functional sensory unit. This structure provides the anatomical basis for the realization of bidirectional communication and functional interaction between neurons and SGCs, thereby enabling SGC-mediated regulation of synaptic plasticity (109).

Studies have shown that purified IgG extracted from FMS patients with FMS specifically binds to rat dorsal root ganglia (DRG), with nine distinct binding clusters identified, suggesting that autoimmune antibodies may participate in FMS mediated by SCGs (110). Andreas Goebel et al. transferred IgG from patients with FMS into naïve mice, inducing increased sensitivity to noxious mechanical and cold stimulation. This hypersensitivity was accompanied by IgG accumulation of IgG in glial fibrillary acidic protein–positive (GFAP+) satellite glial cells in the DRG, indicating that autoimmune antibodies may bind to GFAP^+^ SGCs to induce NPP (107). Silvia Fanton et al. further reported confirmed a positive correlation between anti-satellite IgG levels and pain intensity in FMS, as well as a negative correlation between anti-satellite IgG levels and concentrations of metabolites such as thalamic scyllo-inositol, total choline, and macromolecule 12 (MM12) (111). Emerson Krock et al. confirmed these findings in human samples and further demonstrated a positive correlation between anti-satellite IgG levels and pain intensity in FMS; however, the mechanisms by which SGCs induce NPP remain unclear (112). Menachem Hanani proposed that IgG may induce NPP by binding to and activating SGCs, thereby promoting the release of pro-inflammatory cytokines (113). Jenny E. Jakobsson et al. reported that FMS symptom severity was positively associated with lysophosphatidylcholines (LPCs), suggesting that FMS may be related to altered lipid metabolism (114). IgG complexes binding to Fcγ receptors expressed on DRG neurons can evoke nociceptive activity in the absence of overt inflammation (105, 115). Moreover, there are several shared biomarkers, including calcitonin gene-related peptide (CGRP) receptors, are expressed on both SGCs and DRG neurons. Thus, SGC–sensory neuron interactions mediated by CGRP/CGRP receptor signaling may represent a potential mechanism underlying NPP development (116).

## 7 Central glial cells contribute to NPP development

Concomitant activation of microglia and astrocytes observed in a reserpine-induced FMS rat model underscores the pivotal role of central glial cell activation in NPP (117). Intracerebral administration of minocycline to deplete activated microglia attenuated generalized pain in a repeated acid saline–induced fibromyalgia-like model (88). Similarly, inhibition of astrocytic activation in the spinal cord and prefrontal cortex using Boswellia serrata attenuated pain hypersensitivity was attenuated in FMS model rats (118). Collectively, these studies indicate that activation of central glial cells activation plays a critical role in NPP development. In addition to activation, the polarization of central glial cells has also been reported to contribute to NPP pathogenesis of NPP (119). Multiple studies have demonstrated that central glial cell polarization is involved in NPP. In a reserpine-induced FMS model, microglia rapidly shifted toward the M1 phenotype, resulting in an increased M1/M2 ratio. This shift was further evidenced by a nearly fourfold upregulation of the M1-associated marker inducible nitric oxide synthase (iNOS) and fivefold downregulation of the M2-associated marker arginase-1 (Arg-1) compared with controls (120, 121). Consistently, in a CRPS model, the spinal cord showed increased populations of reactive astrocytes and microglia were observed in the spinal cord, along with elevated expression of the M1 marker CD86 and reduced Arg-1 expression, indicating a preferential M1 microglial polarization in NPP (122). However, the precise mechanisms by which central glial cells mediate the formation of NPP-related chronic pain remain unclear.

In 2022, a study investigating cortical morphological remodeling in patients with CPLBP and FMS revealed that NPP may be associated with structural remodeling of the myelinated architecture in the limbic system cortex. This remodeling was accompanied by upregulated expression patterns in microglia, astrocytes, and oligodendrocyte precursor cells, suggesting that plasticity mediated by central glial cells may represent an important mechanism underlying NPP (123).

### 7.1 Central glial cells modulate synaptic plasticity modulation

Astrocytes, together with microglia, are structurally connected to synapses via gap junctions and form a structural barrier surrounding synapses. This arrangement not only prevents glutamate (Glu) leakage of Glu, but also facilitates the exchange function of ions, neurotransmitters, and other small molecules (124, 125). These cells bear the burden of supporting the basic metabolic requirements of neurons. Highly plastic glial cells not only mediate central immune responses and regulate central infections but also exert multiple functions involved in chronic pain modulation, such as phagocytosis of cellular debris and regulation of neuronal development (126). In parallel, this pathological basis not only provides a route for material and information transfer between neurons and establishes a structural foundation for glial cell–mediated synaptic plasticity changes mediated by glial cells that modulate central sensitization.

Recently, numerous studies have reported that NPP formation appears to be associated with synaptic plasticity changes mediated by central glial cells. A neuroimaging study investigating glial cell activation in patients with FMS revealed widespread cortical thickening compared with healthy controls, a phenomenon particularly prominent in the medial and lateral walls of the frontal and parietal lobes. Concurrent imaging data indicated that microglia play a predominant role in this process, suggesting that cortical plasticity occurs in the brains of FMS patients with FMS and is primarily mediated by microglial activation (127). In rat models of chronic abdominal pain (CAP), microglia-mediated engulfment of postsynaptic spines was increased (128). Application of a low-dose microglial inhibitor, minocycline, can dose-dependently enhanced long-term potentiation (LTP) effect of type C nerve fibers in the spinal dorsal horn induced by high-frequency electrical stimulation, whereas high-dose of minocycline reversed this long-term enhancement effect of type C nerve fibers in the spinal dorsal horn induced by high-frequency electrical stimulation to long-term inhibition (129, 130). These findings indicate that microglia can regulate synaptic plasticity and participate in central sensitization. Alterations in synaptic plasticity may represent a common potential pathological mechanism underlying both neuropathic pain and NPP.

### 7.2 Signaling pathway driven-activation (polarization) of central glial cells

Mitogen-activated protein kinases (MAPK) signaling pathway: Activation of central glial cells is primarily characterized by morphological changes, upregulation of classical activation markers, expansion of cell populations, and enhanced pro-inflammatory responses. Among the signaling pathways involved, MAPK activation represents a key mechanism through which microglia regulate synaptic plasticity. This pathway comprises three major branches: extracellular signal-regulated kinase (ERK), p38, and c-Jun N-terminal kinase (JNK). Increased phosphorylation of ERK, p38, and JNK has been observed in microglia from FMS model animals. Attenuation of pain hypersensitivity following inhibition of these respective phosphorylation events—achieved through specific inhibitors or acupuncture treatment—suggests that the MAPK signaling pathway is a critical mechanism through which microglia mediate NPP (131, 132).

Specifically, the ERK/MAPK pathway is mainly responsible for mediating microglial proliferation and associated morphological alterations. In general, the specific transformations observed in activated states are characterized by astrocytic hypertrophy with elongated and thickened processes, alongside microglial soma enlargement with shortened or retracted processes that may adopt an amoeboid morphology. These changes are structurally conducive to modifying the glia–neuron contact areas (12). However, the morphological alterations alone may not definitively indicate functional activation. Instead, these adaptations provide an anatomical substrate for subsequent alterations in synaptic plasticity changes. In the spinal cord of a mouse model of FMS induced by repeated cold stress (RCS), neuronal hyperactivation was accompanied by the activation and proliferation of surrounding microglia. Depletion of microglia using PLX3397 (pexidartinib) alleviated RCS-induced hyperalgesia, indicating that glial cell activation and excessive proliferation represent a critical pathway in NPP development of NPP (133). Although not directly demonstrated, we propose a hypothesis that ERK signaling pathway–mediated glial cell proliferation may contribute to NPP pathogenesis of NPP. While microglial proliferation has been observed in M1-mediated CRPS models, increases in phosphorylated ERK1/2 (Tyr204)/ERK1/2 failed to reach statistical significance in some studies (122). We suggest that this discrepancy may result from limited sampling in Western blot analyses, given the established role of phosphorylated ERK phosphorylation in mediating microglial proliferation and morphological remodeling.

Other signaling pathways associated with MAPK: The JAK/STAT signaling pathway has also been implicated in NPP. In CRPS, IL-6 signaling via two primary cascades promotes neuroinflammation and pain through two primary cascades: one involving SRC/JAK–STAT activation leading to PTGS2 upregulation and prostaglandin E2 (PGE2) release, and the other proceeding via a PTN11-dependent RasGTPase/MAPK/CEBPB pathway. In addition, tumor necrosis factor (TNF) binding to TNFRSF1B activates TRAF2/MAPK signaling, enhances TRPV1 and SCN10A expression, and thereby facilitates NPP development in sequence (134). Beyond MAPK-mediated pathways, suppression of the α7 nicotinic acetylcholine receptor (α7-nAChR)–dependent cAMP/PKA and PI3K/AKT signaling pathways has also been implicated in NPP pathogenesis of NPP. In a reserpine-induced FMS model, the suppression of the α7-nAChR/PI3K/AKT/Nrf2/Bcl-2 signaling pathway in spinal microglia led to reduced expression of Nrf2, superoxide dismutase (SOD), glutathione (GSH), and Bcl-2. Additionally, reserpine injection inhibited the expression of PKA, phosphorylated AKT1, and phosphorylated CREB. Specifically, suppression of the α7-nAChR/cAMP/PKA/CREB signaling pathway, reserpine promoted microglial polarization of microglia toward the M1 phenotype and induced neuronal apoptosis, thereby altering neural plasticity (120).Activation of Nrf2 acts as a critical bridge for inhibiting the p38 MAPK pathway, while CREB activation competes with NF-κB for limited coactivators, thereby suppressing the expression of pro-inflammatory gene expression. Consequently, the suppression of both the α7-nAChR/PI3K/AKT/Nrf2/Bcl-2 and α7-nAChR/cAMP/PKA/CREB pathways synergizes with p38 MAPK activation to promote central sensitization.

## 8 Cellular basis of synaptic plasticity mediated by activated non-neuronal cells

### 8.1 Pro-inflammatory cytokines

Pro-inflammatory cytokines (PICs), such as interleukin-1β (IL-1β), interleukin-6 (IL-6), and tumor necrosis factor -α (TNF-α), have been reported to be positively associated with NPP. Krisztina Pohóczky et al. performed unbiased transcriptomic analysis of the dorsal root ganglia (DRG) to identify differentially expressed genes in CRPS model mice and found that TNF and Janus kinase (JAK)/STAT) signaling pathways may be involved in CRPS development. Treatment with the soluble TNF-α receptor etanercept or the JAK inhibitor tofacitinib reversed CRPS IgG–induced hyperalgesia and microglial activation were reversed (134). Zsuzsanna Helyes et al. found that blockade of IL-1 successfully reversed profound glial activation and allodynia in naïve mice receiving adoptive transfer of purified IgG isolated from CRPS model mice, indicating that IL-1 also plays a key role in NPP development (135). Masahiro Ohgidani et al. generated human-induced microglia-like (iMG) cells from FMS patients with FMS and exposed them to ATP *in vitro*, identifying a moderate correlation between NPP and ATP-induced upregulation of TNF-α expression (136). Although previous studies have demonstrated intrinsic associations between PICs and NPP in multiple animal models, further investigation is required to clarify how PICs regulate NPP through synaptic plasticity.

Mechanistically, PICs can directly enhance central sensitization by increasing excitatory synaptic transmission or suppressing inhibitory synaptic transmission. Studies have shown that PICs can directly bind directly to glutamate receptors on neurons, thereby enhancing synaptic excitability, and promoting both central sensitization and NPP development (137). Pretreatment with IL-1β and TNF-α increased the frequency of spontaneous excitatory postsynaptic currents (sEPSCs) in lamina II neurons of isolated spinal cord preparations. Meanwhile, IL-1β and IL-6 exposure enhanced both γ-aminobutyric acid (GABA)- and glutamate-induced currents, respectively. Interestingly, IL-6 exposure reduced the frequency of spontaneous inhibitory postsynaptic currents (sIPSCs), while soluble IL-6 initially decreased sEPSCs, followed by increased sEPSCs and cAMP response element-binding protein (CREB) phosphorylation (138).

Beyond direct regulation of neuronal excitability via synaptic plasticity, PICs can also enhance neuronal excitability by activating glial cells and promoting A1 (astrocytic) or M1 (microglial) polarization, leading to further release of pro-inflammatory cytokines. Activated glial cells can secrete the excitatory neurotransmitter glutamate, which binds to N-methyl-D-aspartate receptors (NMDARs) on presynaptic membranes, thereby elevating neuronal excitability (139, 140). Further studies have shown that postsynaptic NMDARs are activated by glutamate released from presynaptic terminals, promoting conversion of arginine to nitric oxide (NO) through a Ca^2+^-dependent pathway catalyzed by inducible nitric oxide synthase (iNOS). Released NO then acts as a retrograde messenger, amplifying the pain signaling through sustained positive feedback regulation of synaptic plasticity (141). Consistently, elevated plasma NO levels have been detected in patients with FMS and migraine and appear to be positively correlated with NMDAR activation of NMDA receptors (142, 143). Collectively, these mechanisms illustrate how PICs and glial cells polarization regulate central sensitization through modulation of synaptic plasticity.

### 8.2 Adenosine triphosphate

The P2X receptor (P2XR) family, including P2X1–P2X7, serves as the receptor of ATP receptors. P2XR subtypes are widely and heterogeneously expressed in the central nervous system (144). Cortical astrocytes express mRNA for P2X1–P2X7, while microglia predominantly express both P2X4 and P2X7. This heterogeneous distribution of P2XR subtypes provides a basis for neuroimmune interactions involved in NPP regulation of NPP (145). Activation of P2X7 promotes astrocyte differentiation, and astrocytes can secrete D-serine, a co-agonist at NMDARs, to enhance both homo- and heterosynaptic long-term potentiation (LTP (146)). Interestingly, P2X2Rs were observed to combine with GABAARs in intracellular directly, activating P2X2Rs could enhance the mobility and degradation of GABA(A)Rs, which might be another potential mechanism for synaptic plasticity changes via purinergic signaling (147). In addition, ATP, a ligand of P2XRs, being released from primary afferent neurons can activate glial cells via C-fiber stimulation, while activated glial cells—particularly microglia—can induce LTP (148–150). Conversely, stimulation of Aβ fibers stimulation can release ATP that activates P2X7 receptors expressed on astrocytes. Activated astrocytes subsequently release ATP, generating synaptic long-term depression (LTD) in neurokinin-1 receptor–positive (NK1R^+^) neurons, leading to antinociception. This divergent effect may reflect astrocytic heterogeneity in terms of morphology and function (151). However, whether distinct astrocyte subtypes regulate NPP through differential synaptic plasticity changes mediated by modulating purinergic signaling pathways remains to be further clarified.

### 8.3 Endogenous cannabinoids

Cannabinoid receptor 1 (CB1) is predominantly distributed in the nerve cells of both the CNS and PNS and regulates both the sensory and affective components of pain, while cannabinoid receptor 2 (CB2) is expressed mainly on immune cells, including central glial cells and peripheral immune cells (152). Ethan B. Russo regarded the formation of NP, represented by FMS, migraine, and IBS, as a deficiency of endogenous cannabinoids; however, whether immune cells could exert regulatory effects on NP through CB-dependent pathways remains unclear (153).

Microglia are a core source of endocannabinoids, while endogenous cannabinoids can produce endocannabinoids anandamide (AEA) and 2-arachidonoylglycerol (2-AG) are produced via N-acyl-phosphatidylethanolamine-phospholipase D (NAPE-PLD)– and diacylglycerol lipase (DAGL)–dependent pathways, respectively. AEA and 2-AG can bind to cannabinoid receptors CB1 and CB2 (154). Activation of CB1 suppresses neuronal activity by reducing Ca^2+^ entry via Cav2.2 channels and potassium efflux through KCC2 following presynaptic vesicle release (155). Activated microglia upregulate CB2 expression of CB2, which promotes transformation toward an anti-inflammatory phenotype, restricts microglial activity to the ipsilateral dorsal horn, and induces release of anti-inflammatory cytokines such as IL-10, thereby alleviating NPP (156).

### 8.4 BDNF

Brain-derived neurotrophic factor (BDNF), regarded as a Cl^−^ homeostasis regulator, has been reported to be positively associated with chronic pain. However, whether BDNF was also represents a pathophysiological feature of NPP remains controversial (157). Regarding the relationship between FMS and the level of BDNF or nerve growth factor (NGF) levels, some studies reported no significant differences between FMS and control patients with FMS and controls (158), whereas Amir Hossein Behnoush et al. found that 15 of 20 studies supported increased level of BDNF levels in FMS (159). Notably, these conflicting findings may arise from multiple contributing factors and do not negate the clinical relevance of BDNF. Similar to NGF, BDNF exhibits dynamic changes, and several studies indicate that elevated BDNF levels decrease following successful treatment (e.g., duloxetine), paralleling improvements in pain and depressive symptoms. This suggests that BDNF may reflect disease activity, and its fluctuation may be attributable to differences in population-level disease status (160). Animal studies further support the pathophysiological role of BDNF. Exercise has been shown to alleviate FMS symptoms and cognitive decline by activating specific signaling pathways, including the hippocampal PGC-1α/FNDC5/BDNF axis (161). Recent high-quality meta-analyses, incorporating stricter quality control, tend to support increased peripheral BDNF levels in FMS (159). As a classical neuromodulator, BDNF still remains to be a promising biomarker for aiding diagnosis and evaluating treatment response.

BDNF is widely expressed in neurons, astrocytes, and microglia, among which microglia are regarded as the primary source of BDNF (162). Activated microglia could release BDNF via ATP-gated Ca^2+^ influx mediated by P2X4 receptors, inducing phosphorylation and activation of p38-MAPK (163). BDNF modulates synaptic plasticity through several mechanisms: 1) BDNF promotes dephosphorylation of KCC2 and downregulates KCC2 expression via the TrkB pathway (164). Through activation of downstream Src homology 2 domain-containing transforming protein/FGF receptor substrate 2 (Shc/FRS-2) and phospholipase Cγ- (PLCγ)/cAMP response element-binding protein signaling pathway, BDNF inhibits intracellular Cl^-^ efflux and regulates ionic plasticity (165). 2) BDNF exerts bidirectional regulation of GABAergic transmission by inhibiting GABA signaling in mature GABAergic neurons while enhancing GABA transmission and facilitating LTD in immature GABAergic neurons via the pro-BDNF/p75 neurotrophin receptor (p75(NTR)) pathway (166, 167). 3) BDNF is not only released by glutamatergic presynaptic terminals and also acts as a retrograde messenger to inhibit the excitation of inhibitory dorsal horn neurons from the postsynaptic membrane (168). 4) In the short term, BDNF regulates synaptic plasticity by enhancing postsynaptic NMDAR activity; in the long term, it increases postsynaptic currents by elevating both mEPSC frequency of mEPSCs and intracellular Ca²^+^ concentration mediated by AMPARs (169). 5) BDNF enhances the pain signal transmission in the descending control system (170). 6) Microglia-derived BDNF induces spinal LTP of synaptic transmission (129).

Although microglia clearly regulate synaptic plasticity through the BDNF/TrkB pathway, whether microglia could mediate NPP through the additional mechanisms described above remains to be further elucidated.

### 8.5 Glu

Astrocytes can express glutamine synthetase (GS), which modulates central glutamine (Gln) synthesis and participates in glutamatergic neurotransmission through the Glu–Gln cycle (171). Under stress conditions, norepinephrine levels increase, enhancing synaptic excitability of synapses. Correspondingly, astrocytes will enhance their ability to take up Glu uptake to prevent synaptic overexcitation. This Glu uptake mechanism protects against neurotoxicity caused by excessive synaptic Glu accumulation and postsynaptic overactivation (172). Astrocytes have been implicated in neuropathic pain mediated by the Glu–Gln cycle; however, whether astrocytes can modulate NP through Glu/GluR-mediated synaptic plasticity remains unclear (173).

Studies show that astrocytes can take up Glu through Na^+^-dependent Glu transporters, Cl^-^-dependent transport mechanisms, and Ca²^+^-dependent transport systems (174). Excessive Glu uptake disrupts ionic balance, leading to astrocytic depolarization and influx of extracellular water, transforming the morphology of astrocytes toward the polarized A1 phenotype and promoting the central sensitization and NP (175).

Regarding ionotropic glutamate receptors (iGluRs), Na^+^ influx of Na^+^ and outflow of K^+^ efflux increase the extracellular K^+^ concentration, promote presynaptic depolarization of the presynaptic terminal to increase the secretion of Glu, and Glu release, and enhance postsynaptic depolarization. For metabotropic glutamate receptors (mGluRs), Glu activates inositol trisphosphate (IP_3_) via PLC-dependent pathways, increasing intracellular Ca^2+^ levels (176). Ca²^+^ acts as a second messenger regulating the adenylate cyclase/cAMP system, with distinct mGluR subtypes exhibiting heterogeneity in this regulation of this signaling pathway) (177).

**Figure 3:**
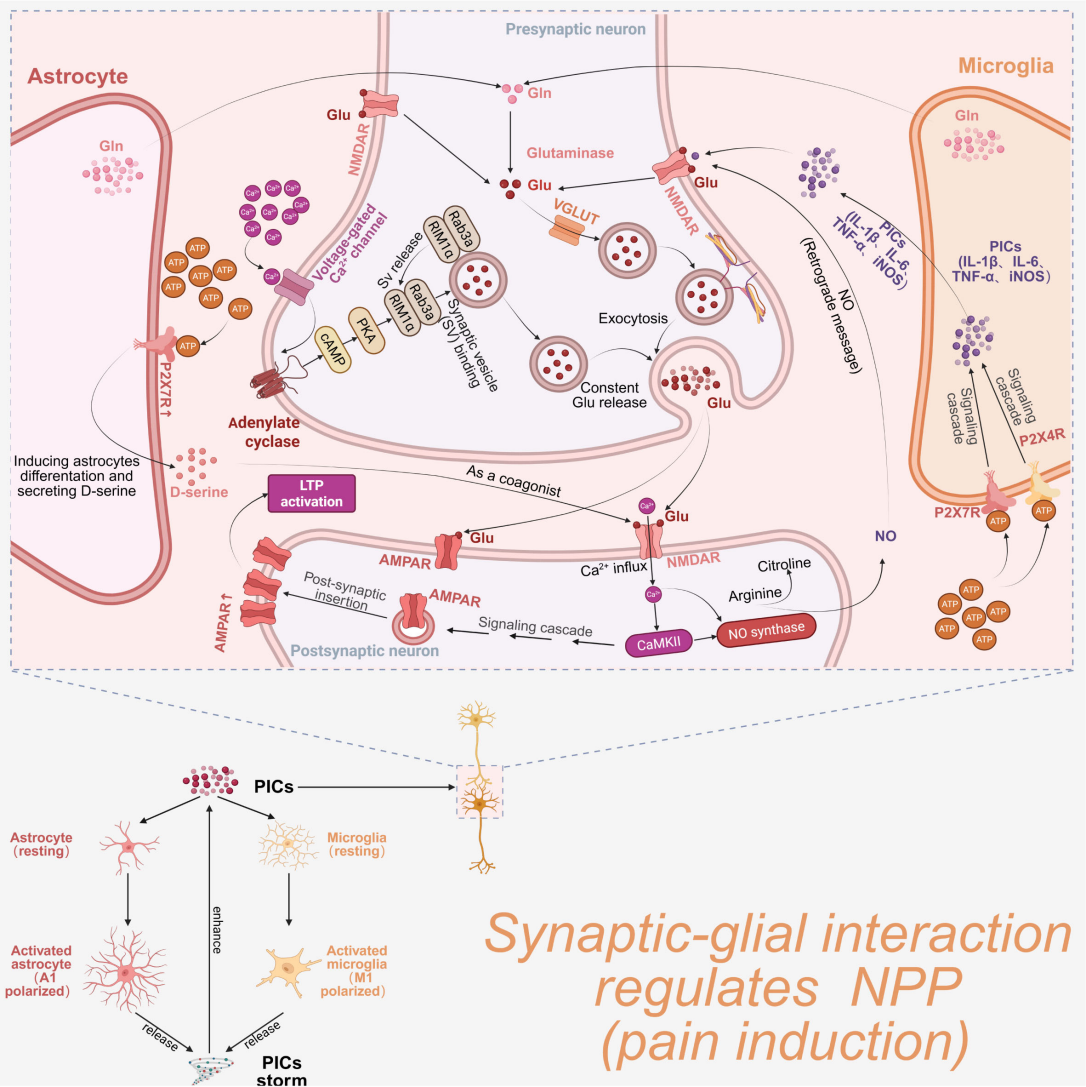
Synaptic–glial interactions promote NPP development. Activated central glial cells, including A1-polarized astrocytes and M1-polarized microglia, can release glutamine (Gln) or modulate the Gln/Glu/VGLUT signaling pathway of presynaptic neurons to promote glutamate (Glu) exocytosis in a voltage-gated Ca^2+^ channel–dependent manner. In the synaptic cleft, accumulated Glu activates AMPARs and NMDARs on postsynaptic neurons to induce long-term potentiation (LTP) or nitric oxide (NO) release NO, respectively. NO acts as a retrograde messenger to further enhance presynaptic neuronal activation.

Additionally, studies have shown that activated immune cells (including central glial cells) can release pro-inflammatory cytokines such as IL-1β, IL-6 and TNF-α. These cytokines can directly bind to glutamate receptors on neurons, enhancing synaptic excitability, and promoting central sensitization and NP development (137). This potential mechanism helps explain how astrocytes and neurons, particularly via synaptic interactions, contribute to NP formation through central sensitization (**Figure 3**).

### 8.6 GABA and glycine

γ-Aminobutyric acid (GABA) is a major inhibitory neurotransmitter acting on GABA receptors (GABA-AR, GABA_BR, and GABA_CR) on the presynaptic membrane or postsynaptic membranes to inhibit synaptic signal transmission and neuronal excitation (178). In the CNS, glial cells regulate nutrient delivery, neurotransmitter interactions, and ionic homeostasis. The “GABA–Glu–Gln cycle enables glial regulation of GABA homeostasis: extracellular GABA enters cells via Glu transporters, where it is converted to Glu by γ-aminobutyric acid aminotransferase, and subsequently synthesized into Gln by GS (179). This process prevents the excessive GABA accumulation, avoiding excessive synaptic inhibition and reducing chronic pain.

Microglia-derived IL-1β can self-activate toll-like receptor 4 (TLR4), thereby reducing presynaptic GABA synthesis, and can activate protein kinase C (PKC) in neurons to suppress postsynaptic GABA receptor activity (180). However, most studies have focused on neuropathic pain, and it is still not clear whether immune cells could mediate the release of GABA to regulate NP via GABA release remains unclear.

In line with the roles of GABA and GABARs, neurotransmitter Gly and its receptor GlyR is the other couple of typical signaling pathway that can inhibit synaptic excitability. Study showed that GABAergic neurons, glutamatergic neurons, and mixed GABAergic/glutamatergic neurons form an interconnected inhibitory neural network. Together with GABA-ARs, GlyRs and the synaptic scaffold protein gephyrin jointly constitute inhibitory synapses that suppress neural excitability (181).

Some terminals co-release both Gly and GABA via vesicles with the assistance of vesicular inhibitory amino acid transporter (VIAAT), exerting analgesic effects (182). Inhibition of glycinergic/GABAergic synapses disrupts descending inhibitory control, enhances hyperalgesia, and induces central sensitization (183). Pharmacological blockade of GABA-ARs and GlyRs allows polysynaptic A-fiber input to NK1R-expressing projection neurons expressing NK1R become obvious, indicating that under disinhibition, non-noxious mechanical stimulation can activate the pain pathways and induce hyperalgesia. While this clarifies how glycinergic neurons contribute to NPP, the role of non-neuronal cells remains unclear (184).

**Figure 4:**
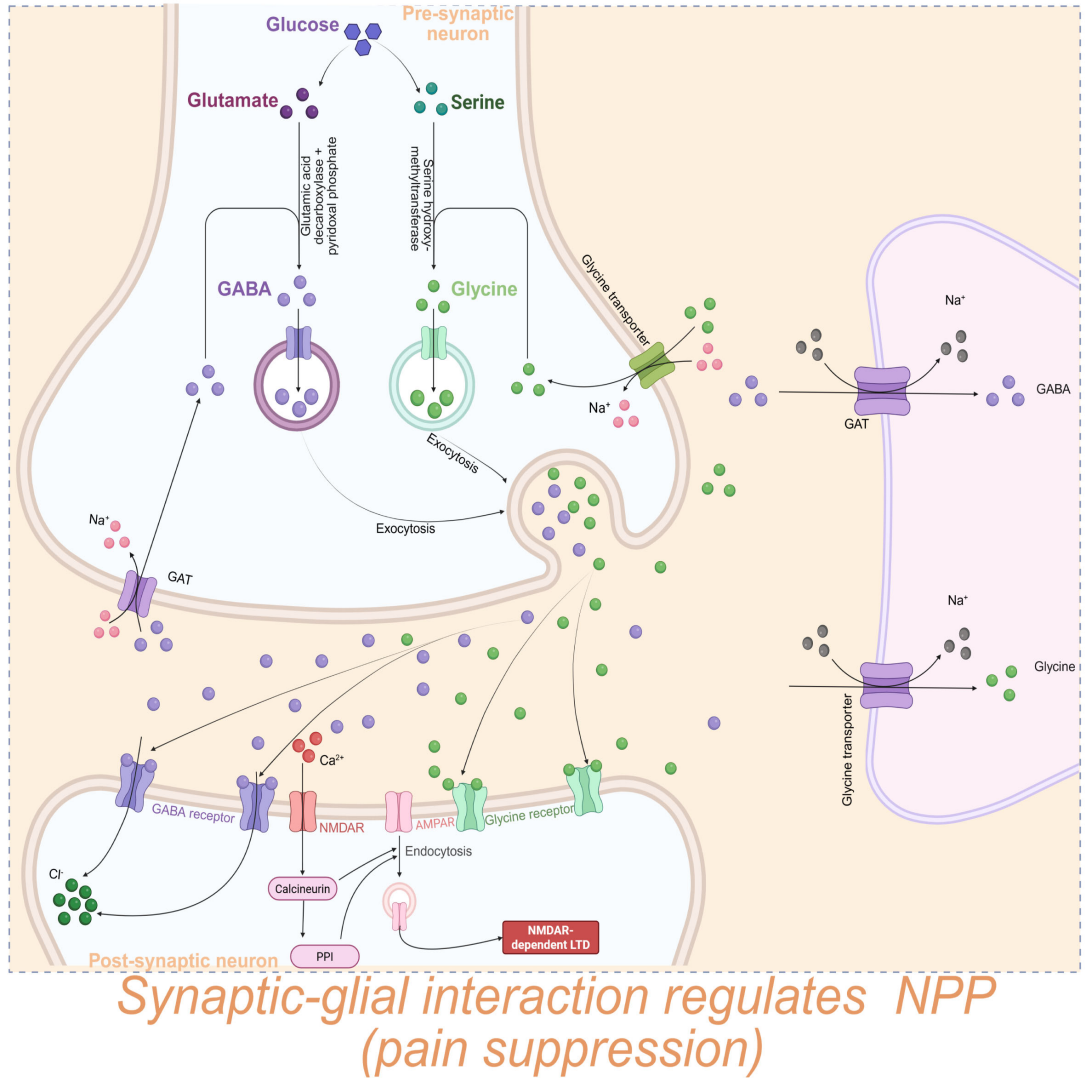
Synaptic–glial interactions inhibit NPP development through GABA/Gly signaling pathways. Central glial cells (on the right, the purple) could generate GABA and glycine (Gly) via GABA transporters or glycine transporters in a Na^+^ dependent manner. Presynaptic neurons could release GABA and Gly into the synaptic cleft through glucose/glutamate/GABA or glucose/Gly pathways, respectively. Accumulated GABA or Gly binds to their respective receptors to generate Cl^-^ influx. In addition, Ca²^+^ binding to NMDARs on postsynaptic neurons induces NMDAR-dependent long-term depression (LTD), thereby inhibiting synaptic excitability and attenuating NPP.

Given that immune cells, especially immune cells, can release IL-1β and prostaglandin E_2_ (PGE2) further studies show that Glyergic synapses exposed to IL-1β exposure induces LTP in glycinergic synapses, while PGE2 selectively inhibits excitatory signals of glycinergic synapses on non-glycinergic neurons, thereby suppressing glycinergic inhibition and promoting chronic pain (185, 186). These findings suggest a potential mechanism by which immune cells mediate NPP formation through glycine-dependent synaptic inhibition (**Figure 4**).

## 9 Modern NPP therapies targeting non-neuronal cells

As summarized in **Tables 2** and **3**, although several drugs for managing NPP have received FDA approval or are recommended by clinical guidelines, most of which are repurposed from other therapeutic areas rather than specifically developed for NPP. These include antidepressants such as duloxetine and milnacipran (serotonin–norepinephrine reuptake inhibitors), tricyclic antidepressants such as amitriptyline, central nervous system depressants such as zolpidem, voltage-gated Ca²^+^ channel inhibitors including pregabalin and gabapentin, muscle relaxants such as cyclobenzaprine orally disintegrating tablets, and opioid analgesics such as tramadol and oxycodone. While these agents may exert analgesic effects—either directly or indirectly—through mechanisms such as inhibition of central sensitization or activation of opioid receptors, they are not specifically designed to target NPP pathophysiology.

**Table 2:** First-line pharmacological agents for NPP management.

Drug	Category	Disease	FDA-approved?	Side effects	Reference (PMID number)
Duloxetine	Anti-depressant: serotonin-norepinephrine reuptake inhibitor	Fibromyalgia	✓	Tachycardia and hypertension	35347488 24385423
Milnacipran	Antidepressant: serotonin-norepinephrine reuptake inhibitor	Fibromyalgia	✓	Nausea, palpitations, headache, fatigue, tachycardia, insomnia, xerostomia, constipation and serotonin syndrome	35587348 26482422 31453551
Pregabalin/Gabapentin	voltage-gated Ca^2+^ channel inhibitor	Fibromyalgia, Chronic Low Back Pain	Fibromyalgia:✓ Chronic Low Back Pain:×	Dizziness, somnolence, headache, and edema	21146466 28045473 41239760
Amitriptyline	Tricyclic antidepressant	Fibromyalgia, Irritable Bowel Syndrome, Chronic Low Back Pain	Fibromyalgia:✓ Irritable Bowel Syndrome:× Chronic Low Back Pain:×	Tachyphylaxis and cardiotoxic effects, dry mouth, sedation, constipation	26395929 37165930 31453551 26820898
Cyclobenzaprine(sublingual tablets formulation——TONMYA	Muscle relaxant	Fibromyalgia	×	Dry mouth, dizziness, fatigue, constipation, drowsiness, nausea, dyspepsia	27377815 38428940
Cannabinoids	Cannabinoid receptor agonist	Fibromyalgia	×	Dizziness, drowsiness, hypotension, hypoglyce- mia, disturbed sleep, tachycardia, cardiac palpitations, anxiety, sweating, and psychosis	27101219 31268891 25139817 34567876 41239760
Tramadol	Opioid; Serotonin-norepinephrine reuptake inhibitor	Fibromyalgia	×	Insomnia, rug dependence and withdrawal syndromes	8955860 31799728 31268891 12753877
Oxycodone	Opioid analgesic	Chronic Low Back Pain	✓	Nausea, constipation, dizziness, dependence, respiratory depression	26820898
Naltrexone	Opioid antagonist	Fibromyalgia	×	/	36974308 38258677
Zolpidem	CNS depressant	Fibromyalgia	×	Dizziness, headache, somnolence, confusion, agitation, abdominal pain, constipation, and xerostomia	33024295
Rifaximin	Antibiotic	Irritable Bowel Syndrome	✓	Nausea, flatulence	39835671
Dicyclomine/ Pinaverium	Antispasmodics	Irritable Bowel Syndrome	✓	Dry mouth, blurred vision	39835671
Linaclotide	Guanylate cyclase-C agonist	Irritable Bowel Syndrome	✓	Diarrhea	39835671
Alosetron	5-HT3 receptor antagonist	Irritable Bowel Syndrome	✓	Constipation, ischemic colitis	17488783
Ibuprofen	Non-steroidal anti-inflammatory drug	Chronic low back pain	✓	Gastrointestinal discomfort, bleeding risk, and nephrotoxicity	26820898

**Table 3:** Commonly used preclinical models and pharmacotherapies for NPP.

Disease	Model	species	Treatment	Reference (PMID number)
fibromyalgia	Reserpin-induced fibromyalgia model	Rattus norvegicus	Salmeterol (β2 agonist)	39496328
		Rattus norvegicus	Myricitrin	39059685
		Rattus norvegicus	Vanillin/Milnacipran	39924579
		Rattus norvegicus	Boswellia	40997946
		Mus musculus	Astaxanthin	38852510
		Mus musculus	Ashwagandha	41002443
		Mus musculus	Eugenia Brasiliensis Leaves	38639862
	Intermittent psychological stress-induced generalized pain model	Mus musculus	Compound A—a lysophosphatidic acid receptor 1 antagonist	40288824
	Acid saline-induced fibromyalgia model	Rattus norvegicus	Tryptophan	39404410
	Intermittent cold stress-induced fibromyalgia model	Mus musculus	Valeriana fauriei	29115388
Irritable Bowel Syndrome	High-fat diet, restraint stress, and senna gavage induced an irritable bowel syndrome model	Rattus norvegicus	Pomegranate peel extract	39599640
	Repeated water avoidance stress induced an irritable bowel syndrome model	Rattus norvegicus	STW 5-II	39454377
	Zymosan-induced irritable bowel syndrome model	Mus musculus	Atractylodes macrocephala Koidz	38892616
	5-hydroxytryptamine-induced irritable bowel syndrome model	Mus musculus	Lysimachia vulgaris var. davurica	38611770
Complex regional pain syndrome	Ischemia-reperfusion injury-induced complex regional pain syndrome type-I/Chronic post-ischemic pain model	Mus musculus	The oleaginous extract of P. pubescens Benth	22728247

Additionally, other medications—including antispasmodics (e.g., dicyclomine and pinaverium), antibiotics such as rifaximin, and guanylate cyclase-C agonists such as linaclotide—are sometimes used to manage NPP-related comorbid symptoms but do not directly alleviate pain (187). Given the heterogeneity of conditions underlying NPP, there is remains a notable scarcity of therapeutics that directly target non-neuronal cells implicated in NPP mechanisms, with cannabinoid receptor agonists being one of the few exceptions.

Currently, a number of non-pharmacological therapies have gradually emerged in clinical practice for the treatment of NPP, such as transcranial direct current stimulation, mindfulness-based therapy, meditation therapy, and physical exercise, all of which have demonstrated favorable therapeutic outcomes (188, 189).

In addition, several ethnopharmacological agents and their active constituents—such as myricitrin, Boswellia, and Ashwagandha—have been increasingly utilized in the treatment of NPP (as shown in **Table 3**) (190–192). However, while existing research acknowledges the involvement of non-neuronal cells, particularly microglia, the underlying mechanisms remain inadequately elucidated. There is still a lack of in-depth investigation into the interactions between non-neuronal cells and neurons. Therefore, this review aims to identify potential targets and clarify underlying mechanisms of NPP, which may facilitate the development of novel, mechanism-based therapies for this complex pain disorder.

## 10 Limitations

Compared with NCP or NP, NPP is a relatively new field, and to date there have been few high-quality studies or groundbreaking advances in either basic or clinical research. In the databases currently available to us, the quality of relevant literature remains relatively low, which may be attributed to insufficient attention given to NPP by researchers. Therefore, while striving to comprehensively collect and incorporate existing literature—particularly by screening more recent publications—we have aimed to provide a broad and novel perspective on the potential mechanisms by which non-neuronal cells mediate the pathogenesis of NPP. Through this approach, we hope to draw greater research attention to the relevance of NPP in neuro-immune interactions, particularly the role of non-neuronal cells, and to highlight potential therapeutic targets, thereby laying a foundation for further in-depth investigation.

In this review, we have incorporated content related to potential biomarkers for NPP, offering new perspectives for the clinical auxiliary clinical diagnosis of NPP. However, it should be noted that NPP is not a single disease but rather a novel mechanistic concept that may contribute to various conditions, such as FMS, CRPS, and IBS. Common pathogenic features of NPP include peripheral and central sensitization, making it difficult to establish diagnostic algorithms based on any single specific biomarker. To date, there have been no high-quality clinical disease modeling or prediction studies specifically focused on NPP. Our work is also constrained by challenges such as limited clinical sample availability and the generally low quality of currently published NPP literature, which hampers the ability to conduct corresponding original research.

As a common biochemical parameter, blood glucose levels have been widely reported to be associated with NPP. However, their specificity is questionable, as glucose levels are highly variable and influenced by factors such as diet, acute stress, and cortisol (193). Thus, claiming blood glucose as a predictive indicator for NPP may be overstated without acknowledging its limited specificity. Similarly, the neutrophil-to-lymphocyte ratio (NLR), a widely used marker, shows substantial elevation in conditions such as tumors and inflammatory diseases. Although NLR demonstrates adequate sensitivity, it also exhibits poor specificity and is influenced by the disease activity in FMS (68). Therefore, these biochemical indicators should be regarded as adjunctive diagnostic tools rather than definitive biomarkers for NPP.

## 11 Discussions and future perspectives

NPP, another type of chronic pain distinct from nociceptive pain and neuropathic pain, encompasses several intractable pain disorders such as FMS, CRPS, IBD, and others (**Table 4**). Studies have shown that different NPP-related diseases can transform into each other. For example, 28–59% of patients with FMS that develop IBS, and 32–77% of patients diagnosed with the diagnosis of IBS develop FMS during the course of their illness, with a relative risk increase of approximately 1.54-fold. These findings suggest a bidirectional correlation between these conditions (194). Owing to their similar pathogenic mechanisms, different types of NPP-related diseases may also transform into one another, providing new perspectives for the treatment of disorders such as IBS and FMS.

**Table 4:** Characteristics of preclinical models for NP and NPP and their corresponding potential targets.

Category	Diseases	Typical model	Behavioral characteristics	Biochemical markers/Inner mechanism	Reference (PMID)
NPP	Fibromyalgia	Reserpine-induced fibromyalgia model	Chronic Widespread Pain Depressive-like Comorbidity	The levels of dopamine, norepinephrine, and serotonin in the spinal cord, thalamus, and prefrontal cortex↓	19646816
		Acid saline-induced fibromyalgia model	A persistent (30-day), bilateral mechanical hyperalgesia, without heat hyperalgesia	The activation of the ASIC3 channel expressed on primary afferent fibers↑	11150964 14659506
		Intermittent cold stress-induced fibromyalgia model/Intermittent cold stress-induced fibromyalgia model/repeated cold stress.	Widespread Musculoskeletal Pain Fatigue Depression	ATP levels↓ Energy metabolism markers (COQ10B, COX4I1, LDH)↑ Glycogen stores↓ Caspase-3 activity↓	18990235 38255163
	Irritable Bowel Syndrome	Maternal separation -induced irritable bowel syndrome model	Visceral hypersensitivity and depressive-like state in adulthood	The levels of corticotropin-releasing factor in serum↑ Gut microbiota dysbiosis (e.g., altered proportions of clostridium clusters XI, XIVa, and XVIII ) The level of 1-Methylnicotinamide in both fecal and serum↑	11804852 36889449
		Rectal enemas of butyrate induced a colonic hypersensitivity model	Decreasing the visceral pain threshold	The c-Fos expression in the thoracic (T10-T12) segments↑ The ASIC1 and ASIC2 mRNAs levels in the lumbar spinal cord neurons↑	20888277
		Wrap restraint stress-induced irritable bowel syndrome model	Gastric emptying was not affected, small intestinal transit was inhibited, and large intestinal transit was stimulated by stress, along with an increase in fecal excretion	The levels of adrenocorticotropic hormone and beta-endorphin in plasma↑	2828144
		Colorectal distension -induced irritable bowel syndrome model	Persistent visceral hypersensitivity, depression-like behaviors, and spatial learning impairment	Microglia activation along with PICs release↑ The expression of glucocorticoid receptor in hippocampal↑	26656865
	Complex regional pain syndrome	Tibial fracture/cast immobilization model	Acute Phase (4 weeks): hindpaw allodynia, limb unweighting, warmth, edema, and epidermal thickening Chronic Phase (16 weeks): Transition to a cold, dystrophic limb where only allodynia and unweighting persist	Peripheral(Skin/Nerve): Acute elevation of Substance P, NK1 receptor, PICs, and NGF, which resolve in the chronic phase Central (Spinal Cord): Sustained upregulation of NK1 receptor, TNF-α, IL-1β, and NGF in both acute and chronic phases	26785976
		Chronic post-ischemic pain	Persistent spontaneous pain and hypersensitivity (lasts for 4 weeks) Early manifestations (hours to days post-reperfusion) include hindpaw edema, cyanosis (bluish discoloration during ischemia), followed by subsequent erythema (redness) and increased temperature (during reperfusion)	The expression of ROS and PICs↑ Pro-inflammatory macrophage infiltration in local DRG↑	39182734 40749060 21795964 26490459 15494189
NP	/	Spinal nerve ligation	Having a greater degree of mechanical allodynia	The expression of ADAM8 and ATF-3 in DRG↑ Norepinephrine release↑ 38 proteins, such as synapsin 1 and microtubule-associated protein 2 miR-96↑, miR-182↑, miR-183↑	19442185 40688684 40349732 19358840 25119878
	/	Partial nerve ligation	Spontaneous pain Mechanical allodynia Thermal hyperalgesia Bilateral mechanical hyperalgesia	Neurofilament light chain release in serum↑	19442185
	/	Chronic constriction injury	Having a greater degree of thermal hyperalgesia and cold allodynia	p38 MAPK signaling pathway in hyperactive microglia in the dorsal horn of spinal cord↑	19442185 30762024 18191894
	/	Chemotherapy-induced neuropathic pain	The pain is often resistant to standard analgesics Glove-and-stocking anesthesia	1)Gene variations in glutathione-S-transferase P1, glutathione-S-transferase M1, ERG2, SCN4A, SCN9A, and SCN10A 2)Sodium channel changes in DRG: CaV2.2↑,TRPA1↑,TRPV1↑,TRPM8↑,CCL2/CCR2↑	19442185 33648782
		Diabetic neuropathy	Mechanical allodynia Glove-and-stocking anesthesia	Blood glucose↑ Methylglyoxal and α-oxoaldehyde↑ Glyoxalase I activity↓ Toll-Like Receptor 4↑ Adiponectin↑ miR-146a↓, miR-125a-5p↑, miR-145-3p↑, miR-99b-5p↑, miR-873-5p↑, miR-518d-3p↑, miR-618↑ Nerve-specific enolase↑ Semaphorin 3A↑	33669048

**Figure 5:**
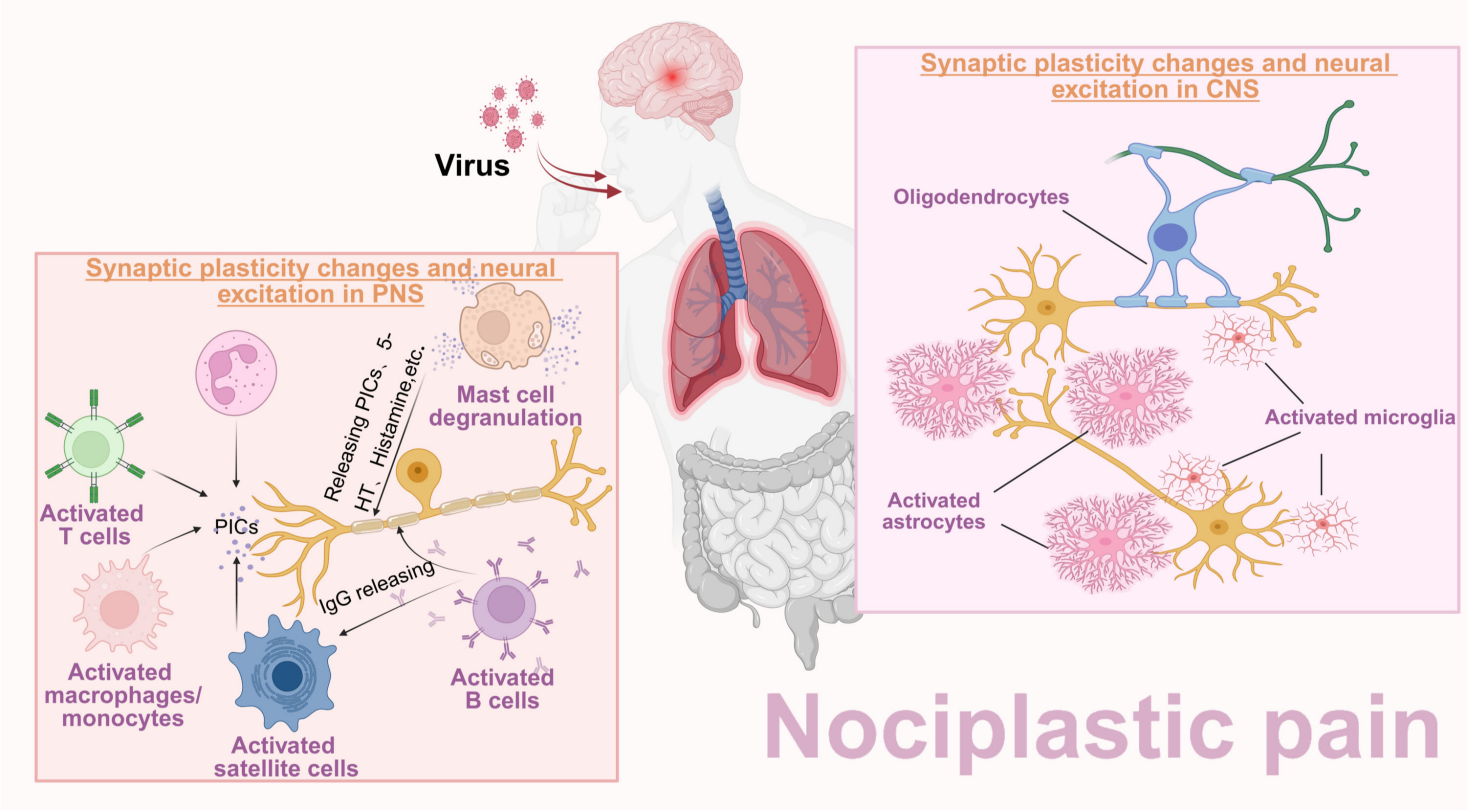
Central sensitization as a shared mechanism of NP and NPP. In NP patients, no matter whether due to peripheral nerve fiber injury or the central nervous system impairment, peripheral sensitization and central sensitization can be induced. In contrast, the underlying mechanism of NPP is mainly attributed to pathogen pre-infection and persistent stimuli of mild central inflammatory stimulation. Both NP and NPP patients share a common mechanism: central sensitization mediated by synaptic plasticity changes and neuronal hyperexcitability.

Essentially, the shared pathogenic core of the pathogenesis of NP and NPP lies in peripheral and/or) central sensitization following repeated and persistent exposure to non-harmful stimuli—that is, a separation of central (or peripheral) sensitization occurring without significant activation of nociceptors or clear evidence of nerve damage of patients (**Figure 5**). Recent studies indicate that the role of non-neuronal cells should not be underestimated, particularly in the context of peripheral and/or) central sensitization. Conventionally, injurious stimuli are often accompanied by inflammation. However, a key unresolved question remains: why do non-neuronal cells, especially the immune cells, mediate pain in the absence of overt tissue damage or inflammation?

Accordingly, this review focuses on non-neuronal cells and aims to systematically illustrate how they participate in the development of NPP. Notably, we propose a hypothesis that pre-infection may represent one of the prerequisite conditions which is essential for the NPP development. Under conditions of prior infection, combined with subsequent stimuli such as light exposure, diet, temperature changes, or even one’s own emotional stress, the NPP may emerge. During the early inflammatory phase of viral infection, viral matrix metalloproteinases and immune-derived cytokines increase blood–brain barrier permeability, facilitating viral entry and immune cell infiltration into the central nervous system. These infiltrating immune cells release PICs that, via MAPK-dominant signaling pathways, induce sustained glial activation. Activated glial cells modulate neuronal excitability through PICs and neurotransmitters such as adenosine. Meanwhile, certain viruses may employ epigenetic mechanisms—such as neuron-specific microRNAs—to suppress transcription factors, promote viral genome heterochromatinization, and induce glial histone methylation, thereby establishing viral latency and altering synaptic gene expression (195, 196). Clinically, late-phase pain often becomes dissociated from inflammation and resistant to anti-inflammatory therapies, resembling NPP. This NPP-like state appears conditional and may transition into a mixed nociceptive–neuropathic–central pain phenotype upon viral reactivation and intense inflammation. However, the viral pre-infection hypothesis is currently not yet fully supported by direct evidence and currently remains primarily a potential, albeit promising, mechanism underlying the pathogenesis of NPP-related chronic pain. Importantly, this hypothesis must be distinguished from ongoing inflammation, small-fiber neuropathy, and residual immune-mediated tissue damage (197–200).

Beyond the central nervous system, the peripheral nervous system—particularly the dorsal root ganglion—may represent another critical site involved in NPP development. Small-fiber neuropathy mediated by excessive activation of sodium channels (Nav1.7), together with sympathetic nerve fiber sprouting induced by nerve growth factor, may help explain why NPP is also regulated by peripheral sensitization (201). In the peripheral nervous system, tissue-resident macrophages and satellite glial cells tightly envelop primary neuronal cell bodies in the form of a network-like structure, while Schwann cells ensheath axons of primary neurons in the form of myelin. Peripheral sensitization is traditionally considered a consequence of nociceptor sensitization induced by peripheral inflammation or peripheral nerve injury mediated by peripheral immune cells.

Interestingly, it has been reported that peripheral immune cells have been reported to cross the blood–brain barrier through their migratory mechanisms and enter the central nervous system to contribute to central sensitization. Therefore, in addition to classical central glial cells, we have summarized various non-neuronal cell types associated with NPP. Although there is no sufficient evidence is lacking to conclusively demonstrate that these related non-neuronal cells can regulate NPP through synaptic plasticity modulation of synaptic plasticity, we propose that they may nonetheless influence NPP through potential mechanisms involving synaptic plasticity regulation.

Finally, NPP remains a relatively new concept that has not yet been comprehensively explored, resulting in a scarcity of directly relevant literature directly addressing the impact of non-neuronal cells on synaptic plasticity in NPP. In this review, we explored potential links between non-neuronal cells, synaptic plasticity, and NPP. Moreover, the regulation of plasticity extends beyond synaptic plasticity alone and should also encompass broader regulatory effects on neuronal function and structural plasticity (**Table 5**). In addition to dysfunctions in plasticity mediated by non-neuronal cells, their structural contributions likewise merit further investigation.

**Table 5:** The contribution of non-neuronal cells to the pathogenesis of NPP.

Diseases	Cell types	Contributions
FMS,CWP	Monocytes/Macrophages	Monocytes/macrophage cell proliferation Releasing PICs Infiltrating into the trigeminal nerve tissues,muscle,DRG, and other spinal cord sites Secrete chemokines to induce other immune cells' migration and infiltration
FMS,IBS	Neutrophils	1) Evaluating NLR 2) Releasing MMP8 3) Infiltrating into the DRG mediated by serum-soluble adhesion molecules
FMS,IBS	Mast Cells	Mast cells migration and local infiltration Mast cells form synaptic-like connections with nerve fibers and influence neurons through the secretion of MCP-1 and N-cadherin, while neurons, in turn, interact through the release of SP, CGRP, and NGF Degranulation releases mediators like histamine and proteases to activate corresponding receptors such as H1R-H3R and PAR2 Activating the 5-HT1A receptor Releasing PICs (e.g., IL-1, IL-18) contributes to pain via the P2X7R/NLRP3 signaling pathway Releasing chemokines to induce other immune cells migration and infiltration
FMS,CRPS	T cells	Altered T-cell subpopulations (e.g., increased CD8+ MAIT cells; decreased CD69^+^ and CD25^+^ T cells; down-regulating CD4^+^FOXP3^+^ T cells) Imbalanced cytokine secretion profile (e.g., elevated IL-17A, IL-22, TNF-α, IFN-γ; Th1/Th2 disproportion) promotes peripheral and central sensitization
FMS,CRPS	B cells	Disordered proportion of B-cell subsets (e.g., increased percentages of MHC class II-positive B cells and memory B cells, decreased percentage of μ-opioid receptor-positive B cells) Release IFN to stimulate DRG neurons via the MNK/eIF4E pathway, causing nociceptive sensitization 3) Produce IgG autoantibodies that bind to satellite glial cells and sensory neurons, inducing hypersensitivity
FMS	Satellite glial cells (SGCs)	IgG antibodies from FMS patients bind to and activate GFAP^+^ satellite cells, prompting pro-inflammatory cytokine release Anti-satellite IgG levels positively correlate with pain intensity and negatively correlate with metabolite concentrations in regions like the thalamus Anti-satellite IgG may directly bind to Fc-γreceptors expressed on DRG neurons to induce NPP Mediate bidirectional communication with sensory neurons via signals like CGRP/CGRP receptors, regulating synaptic plasticity
FMS, CRPS, CAP	Central glial cells (Microglia & Astrocytes)	Central glial cells(especially microglia) activation and polarization (towards M1 microglia and A1 astrocytes) impair synaptic connection via MAPK signaling pathways (ERK, p38, JNK) Release pro-inflammatory cytokines (e.g., TNF-α, IL-1β, IL-6) and bind to GluR on neurons to enhance synaptic excitability (e.g., TNF-α and IL-1β enhance excitatory synaptic transmission by increasing the frequency of spontaneous sEPSCs and amplifying the amplitude of currents evoked by NMDAR activation; IL-6 and IL-1β suppress inhibitory synaptic transmission by reducing the frequency of sIPSCs and diminishing the amplitude of currents induced by GABA or Gly.) Activated glial cells secrete excitatory neurotransmitters such as Glu, which bind to neuronal NMDARs and enhance neuronal excitability Activated microglia-derived IL-1β can bind to its own TLR4 receptors, suppress presynaptic GABA synthesis, and activate neuronal PKC to inhibit postsynaptic GABA receptor activity IL-1β, together with prostaglandin E2 released by glial cells, can inhibit the excitability of glycinergic synapses P2X2Rs expressed on glial cells can directly bind to GABA(A)Rs intracellularly, enhancing the mobility and degradation of GABA(A)Rs Released ATP can activate P2X7Rs, promoting astrocyte differentiation and inducing the secretion of D-serine and the formation of LTP, or alternatively, it can induce LTD in NK1R-positive neurons Microglia-derived endocannabinoids can bind to CB1 via the AEA pathway, inhibiting calcium influx and potassium efflux, thereby suppressing presynaptic vesicle release. Alternatively, they can upregulate CB2 expression via 2-AG, promoting the transition of microglia toward the M2-polarized phenotype BDNF released from microglia activated via the P2X4R/p38MAPK pathway modulates synaptic plasticity through multiple mechanisms: downregulating KCC2 via TrkB to disrupt chloride homeostasis, bidirectionally regulating GABAergic transmission, acting as a retrograde messenger, enhancing postsynaptic excitability via NMDAR/AMPAR-mediated mechanisms, facilitating descending pain pathways, and inducing spinal LTP
